# Pardon the interruption: how Und-P sequestration has reshaped our understanding of the bacterial cell envelope

**DOI:** 10.1128/jb.00451-25

**Published:** 2025-12-18

**Authors:** Matthew A. Jorgenson

**Affiliations:** 1 Department of Microbiology and Immunology, University of Arkansas for Medical Sciences 318223, Little Rock, Arkansas, USA; University of Southern California, Los Angeles, California, USA

**Keywords:** undecaprenyl phosphate, sequestration, polysaccharide, glycan, cell envelope

## Abstract

The bacterial cell envelope is composed of glycans that maintain cell shape and protect against antibiotics, immune systems, and other environmental hazards. The glycans found in the envelopes of most bacteria are assembled on and transported by the essential lipid carrier undecaprenyl phosphate (Und-P). Und-P is activated for glycan assembly by the dephosphorylation of undecaprenyl pyrophosphate (Und-PP), both during *de novo* synthesis and after Und-PP is released from glycan intermediates. Und-P sequestration occurs when Und-P cannot release from glycan precursors or Und-P(P)-binding compounds. Importantly, cells that cannot recycle Und-P produce too few cell wall precursors to sustain growth and will eventually lyse. This review summarizes the Und-P sequestration narrative, from its sporadic observation to its eventual mechanistic understanding. More broadly, deciphering Und-P sequestration has reshaped how we interpret phenotypes, uncover mechanisms of envelope assembly, and determine the activity of antibacterials. The far-reaching implications of these findings suggest that continued study of Und-P sequestration will not only yield a deeper understanding of the bacterial cell envelope but also reveal new strategies to disrupt this protective barrier.

## INTRODUCTION

The bacterial cell envelope is a multilayered structure that surrounds and protects the cytoplasm (reviewed in reference 1). For gram-positive bacteria, the basic configuration consists of a cytoplasmic membrane and a thick peptidoglycan (PG) cell wall containing teichoic acids. The gram-negative envelope is more complex, featuring an inner membrane, a thin PG layer, and an outer membrane (OM) containing lipopolysaccharide (LPS). Bacterial glycans are foundational to envelope assembly and serve as virulence factors for pathogens and as antigens in vaccines. These key molecules are often linked to proteins and lipids, structures collectively known as glycoconjugates (reviewed in reference 2). The glycoconjugates found in the envelopes of gram-positive and gram-negative bacteria display enormous structural diversity and include PG, capsules, exopolysaccharides, LPS O-antigens, teichoic acids, lipoglycans, and many other clinically important polymers. Despite this structural diversity, the glycan moiety of nearly all bacterial glycoconjugates is assembled on and transported by an essential lipid carrier known as undecaprenyl phosphate (Und-P), which is also referred to as bactoprenyl phosphate.

## OVERVIEW OF UND-P BIOSYNTHESIS

**Fig 1:**
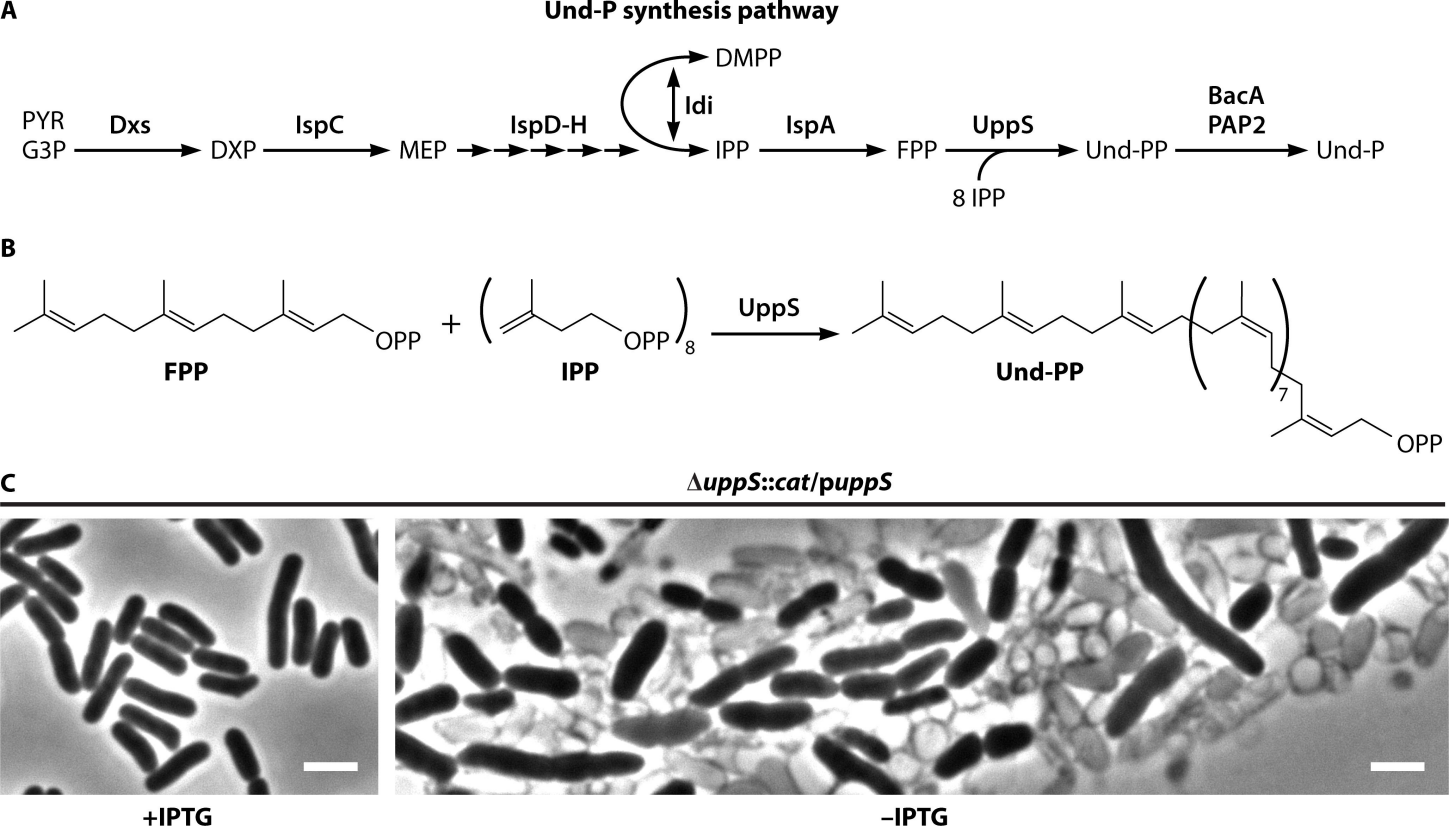
Biosynthesis of Und-P and its essential role in maintaining cell integrity. (**A**) The MEP pathway (Dxs→Idi) generates the isoprenoid precursors that are condensed by UppS to produce Und-PP. Und-P is activated for glycan synthesis by Und-PP phosphatases, which generate the monophosphate linkage. Abbreviations: DMPP, dimethylallyl-diphosphate; DXP, 1-deoxy-D-xylulose-5-phosphate; FPP, farnesyl diphosphate; G3P, glyceraldehyde-3-phosphate; IPP, isopentenyl diphosphate; MEP, 2-C-methyl-D-erythritol 4-phosphate; PYR, pyruvate; Und-PP, undecaprenyl pyrophosphate. (**B**) The UppS synthase condenses eight IPP molecules with a single FPP molecule. (**C**) Micrographs showing depletion of UppS in *Escherichia coli* using a plasmid-based system that expresses *uppS* from an IPTG-inducible promoter. After growing several generations without IPTG, the strain became depleted of UppS (and Und-P) and lysed. Bar, 3 μM.

Und-P belongs to a family of isoprene-containing molecules known as polyprenyl phosphates, which function as essential carrier lipids in all biological kingdoms. In bacteria, Und-P is essential because it is required to assemble the PG layer that protects against osmotically induced lysis (reviewed in reference 3). Structurally, Und-P is a 55-carbon isoprenoid (C_55_-P) that is formed via the methylerythritol phosphate (MEP) pathway (Fig. 1A, Dxs→Idi). Und-P is initially synthesized as undecaprenyl pyrophosphate (Und-PP) by the undecaprenyl pyrophosphate synthase (UppS), which condenses eight isopentenyl pyrophosphate (C_5_-PP) molecules with a single farnesyl pyrophosphate (C_15_-PP) (Fig. 1B and reviewed in reference 4). Virtually every step in the Und-P synthesis pathway is essential and, as shown in Fig. 1C, cells depleted for UppS (and thus Und-P) grow misshapen and lyse. While Und-P predominates in the membranes of bacteria, homologs with shorter chains are utilized by certain species, including C_50_-P in *Mycobacteria* (5, 6) and C_45_-P in *Paracoccus denitrificans* (7). Und-PP is generated on the inner leaflet of the inner membrane (gram-negative bacteria) or the cell membrane (gram-positive bacteria) and flipped to the outer leaflet, where it is dephosphorylated to Und-P by the integral membrane pyrophosphatase BacA (8) and PAP2 family proteins (e.g., YbjG, LpxT, PgpB, and BcrC) (9–11). Following dephosphorylation, Und-P is flipped back to the inner leaflet of the inner (cytoplasmic) membrane by DedA and DUF368 family proteins (12, 13), where it is ready for use in glycan assembly. The flip-flop model is based on the observation that the active sites of BacA and PAP2 proteins are positioned outside the cytoplasm (14, 15). Why cells deliberately synthesize Und-P in the inactive Und-PP form (rather than directly as Und-P) and why Und-PP dephosphorylation is restricted to the outer leaflet of the cytoplasmic membrane remain open questions. To that end, synthesizing Und-P in the inactive Und-PP form may help distinguish Und-P(P) from other lipids, regulate the flow of Und-P into pathways, or act as a feedback control for Und-P synthesis.

**Fig 2:**
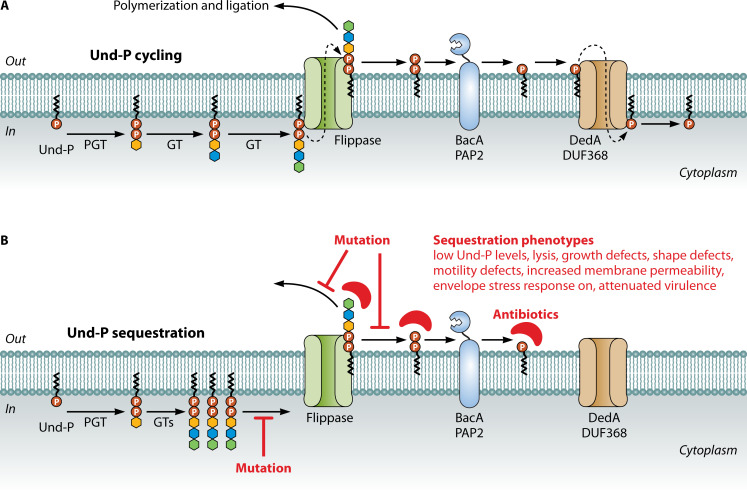
Model for Und-P sequestration. (**A**) General assembly of glycans on Und-P. Phosphoglycosyl transferases (PGTs) transfer phosphosugars from nucleoside diphosphate sugar donors to Und-P to form Und-PP-linked sugar monomers, which are progressively assembled into oligosaccharide building blocks by glycosyltransferases (GTs). Once assembled, Und-PP-linked oligosaccharides are flipped across the inner membrane (gram-negative bacteria) or cell membrane (gram-positive bacteria), where polymerases link them into repetitive glycan polymers. Transfer of glycans to acceptor molecules releases Und-PP, which is dephosphorylated to Und-P by BacA and PAP2 family proteins. Und-P is then flipped to be cytoplasmic facing by DedA and DUF368 family proteins for use in another round of glycan assembly. (**B**) Und-P sequestration. Mutations or antibiotics that sequester Und-P block its recycling, thus reducing the amount of Und-P available for glycan synthesis and creating a wide variety of phenotypes. See Table 3 for the list of antibiotics targeting Und-P(P) and its derivatives.

Once Und-P is facing the cytoplasm, phosphoglycosyl transferases (PGTs) transfer phosphosugars from nucleoside diphosphate sugar donors to the carrier lipid to form Und-PP-linked sugar monomers [reviewed in (16)]. Glycosyltransferases then extend this intermediate until the final membrane-bound unit is formed, which is ultimately flipped across the inner (cytoplasmic) membrane by a dedicated transporter (e.g., Wzx and Wzm/Wzt proteins). Eventually, glycans are transferred to an acceptor molecule (e.g., PG, LPS, and protein), which releases Und-PP to be recycled to the cytoplasm via proteins in the BacA/PAP2 (dephosphorylation) and DedA/DUF368 (translocation) families (12, 13). Figure 2A shows a general model for assembly of Und-P-dependent polymers. While most pathways release Und-PP as a by-product, some release Und-P directly, including the 4-amino-4-deoxy-L-arabinose (L-Ara4N) and teichoic acid modification pathways (17, 18). In either case, the free pool of Und-P (i.e., Und-P molecules not bound to glycan intermediates) is maintained through *de novo* synthesis and recycling. Though Und-P is not consumed, its pool size is limited. Recent measurements indicate that gram-negative bacteria typically harbor around 100,000 molecules of free Und-P (19), with similar levels reported in gram-positive bacteria (20). Since Und-P is made in limiting amounts, replenishing the free pool of Und-P is crucial to sustaining synthesis of Und-P-dependent polymers.

## DEFINING UND-P SEQUESTRATION

For most bacteria, Und-P is used in multiple biosynthetic pathways. For example, in the gram-negative bacterium *Escherichia coli*, Und-P is required to synthesize several polysaccharides, including PG (3), enterobacterial common antigen (ECA) (21), O-antigen (22), colanic acid (23), and possibly osmoregulated periplasmic glucans (24). Und-P is also required to modify lipid A with aminoarabinose (17) and O-antigen with glucose (25). It should be noted that lab strains of *E. coli* usually do not produce O-antigen due to insertions in *wbbL* (K-12 strains) or *wbbD* (B strains) (26, 27). Und-P also plays a central role in gram-positive bacteria, where in *Bacillus subtilis* it supports the synthesis of PG, major and minor forms of wall teichoic acids (WTAs), teichuronic acid, and exopolysaccharides (28, 29). Und-P is additionally required to glycosylate lipoteichoic acid and other surface polymers (30, 31). Because many pathways compete for a common pool of Und-P, increased demand in one pathway may reduce the amount of Und-P available to other pathways. Und-P availability can also decrease if it cannot release from a substrate, a situation known as sequestration. Und-P sequestration occurs when an Und-P pathway is interrupted (i.e., by a pathway mutation), so that Und-P- or Und-PP-linked intermediates accumulate, or when Und-P(P) or Und-P(P)-linked derivatives are bound by antibiotics (see later); both of these events prevent recycling of the carrier lipid (Fig. 2B). Since Und-P is shared, sequestration is expected to reduce its availability to all Und-P-using pathways. However, its most pronounced physiological effects are observed in PG synthesis (e.g., growth reduction, morphological defects, and lysis). Other than relieving the source of disruption, Und-P sequestration can be reversed by blocking initiation of the interrupted pathway (genetically or chemically), thus increasing the free pool of Und-P, or by increasing the competitive advantage of a pathway. Note that Und-P is also sequestered indirectly when Und-PP phosphatase or Und-P transporter activity is impaired (9, 14, 32, 33). However, this mechanism is not discussed in detail here since this review focuses on the mechanism involving the accumulation of Und-P(P)-linked derivatives.

## DEVELOPMENT OF THE UND-P SEQUESTRATION MODEL

The physiological consequences of Und-P sequestration were first described in *Salmonella* Und-P pathway mutants during the 1960s and 1970s. In 1969, Yuasa et al. described an LPS mutant of *Salmonella enterica* serovar Typhimurium LT2 (34), whose O-antigen normally contains four sugars: D-mannose, L-rhamnose, and D-galactose in the main chain and abequose as a side branch sugar. The mutant strain (HN238) could not synthesize the abequose side chain and was noted as “extremely unstable,” giving rise to secondary suppressing mutations that either restored abequose incorporation (revertant) or abolished O-antigen expression by disrupting *wbaP* (formerly *rfbP*), which catalyzes the initial step in O-antigen synthesis (34). Based on these observations, the authors reasoned that the accumulation of incomplete O-antigen intermediates brought about this genetic instability. Since O-antigen was known to be assembled on Und-P (35), the authors surmised that such accumulation could “siphon off a large portion of the P-ACL [Und-P] present in the cell and…possibly impair other processes in which P-ACL plays a catalytic role.” They further stated that “[o]ne such process which may be impaired is the biosynthesis of cell wall peptidoglycan.” Thus, the idea that Und-P sequestration could indirectly affect PG synthesis (and any other Und-P-using pathway) was recognized very early.

Soon after Yuasa et al. reported their findings, competition for Und-P between pathways was observed in gram-positive bacteria. In 1971, Watkinson et al. used membrane preparations from *Streptococcus lactis* (now *Lactococcus lactis*) to monitor competition between PG and WTA synthesis (36). The following year, Anderson et al. extended this methodology to *Bacillus licheniformis* (37). In both cases, WTA synthesis was inhibited by adding more PG precursors to the reaction, by introducing bacitracin (which sequesters Und-PP), or by applying both treatments together. Watkinson et al. recognized that if “undecaprenol phosphate is common to both pathways then such reduction would account for the observed inhibition of teichoic acid synthesis” (36). Anderson et al. concurred, noting that the decrease in WTA synthesis in their system was “an indirect effect due to the depletion of common undecaprenol phosphate from the…accumulation of lipid pyrophosphate in this blocked cycle” (37). Later, in 1985, Masson and Holbein used membrane preparations to investigate competition between PG and capsule synthesis in the gram-negative bacterium *Neisseria meningitidis*. As expected, capsule synthesis was inhibited by adding more PG precursors to the reaction (38). Altogether, results from these *in vitro* studies strongly implied that pathways compete for a common pool of Und-P and that sequestration of Und-P in one pathway can reduce output in another.

## THE ORIGIN OF THE TOXIC INTERMEDIATE HYPOTHESIS

Following reports of O-antigen pathway mutants, phenotypes of ECA pathway mutants in *Salmonella* began to emerge, and their interpretation raised questions about the deleterious nature of Und-PP-linked intermediates. In 1976, Mäkelä et al. analyzed a set of ECA-negative mutants and found that each carried secondary mutations that were likely in *wecA*, which initiates ECA synthesis (39). Interestingly, when the *wecA* suppressor mutations were replaced with wild-type sequence, the resulting recombinants grew poorly (small colony phenotype) and exhibited increased sensitivity to sodium dodecyl sulfate (SDS), indicating an OM permeability defect (39). Secondary mutations in *wecA* also arose in these “corrected” strains when cultured on plates containing SDS (39). Since Und-PP-linked ECA intermediates would not be observed until 1985 (40), the authors generally attributed these phenotypes to the “accumulation of a toxic or otherwise deleterious intermediate,” possibly a sugar (39). This marked one of the earliest instances in which the effects of Und-P sequestration were attributed to a toxic intermediate. In retrospect, the lack of a clear distinction between toxic intermediates and sequestration may explain why Rick et al. did not invoke the sequestration hypothesis when they showed that SDS sensitivity in late-stage ECA pathway mutants was associated with the accumulation of Und-PP-linked ECA intermediates (i.e., ECA lipid II) (41). In any event, with the introduction of the toxic intermediate hypothesis, it became necessary to determine whether Und-P-dependent pathway phenotypes resulted primarily from sequestration or from the buildup of intermediates that were inherently toxic.

To distinguish between these possibilities, a method to increase the cellular pool of Und-P was needed. If phenotypes associated with Und-P pathway mutants could be reversed by increasing Und-P levels, it would mean that sequestration caused those phenotypes since Und-PP-linked intermediates would still accumulate under these conditions. The discovery that *bacA* overexpression confers resistance to bacitracin seemed to offer a solution (42). Since bacitracin sequesters Und-PP, it was thought that bacitracin resistance was mediated by increasing Und-P levels. Based on this reasoning, Danese et al. tested whether *bacA* overexpression could reverse membrane defects in *E. coli* mutants accumulating ECA lipid II (43). When BacA overproduction produced no ameliorative effect on ECA lipid II phenotypes, the authors concluded that it was “unlikely that accumulation of [ECA] lipid II exerts its effects by sequestering C_55_-P [Und-P]” (43). This implied that most, if not all, Und-P(P)-linked intermediates were inherently toxic. It should be noted that no study has shown that *bacA* overexpression increases the free pool of Und-P. In retrospect, direct competition for Und-PP between BacA and bacitracin likely explains why BacA overproduction counteracts bacitracin. In any event, the Danese et al. study helped advance acceptance of the toxic intermediate hypothesis.

## UNIVERSAL CONSEQUENCES FOR A UNIVERSAL LIPID CARRIER

Since Und-P functions as a universal lipid carrier, it follows that many other examples of Und-P sequestration have been documented across bacterial systems. To help convey the extent of this phenomenon, Table 1 presents some key observations of Und-P sequestration in gram-positive and gram-negative systems.

**TABLE 1:** Examples of Und-P sequestration in bacterial systems^*a*^

Organism	Und-P pathway	Evidence for Und-P sequestration
*Salmonella enterica*	O-antigen	In *his-rfb* deletion mutants, in which O-antigen synthesis is interrupted, secondary mutations arose that blocked O-antigen expression (34). Preventing O-antigen initiation by deleting *galE* blunted the deleterious effects of an *abe* null mutation, which disrupts O-antigen expression (44).
	ECA	In long-*his-rfb* deletion mutants, in which ECA synthesis is interrupted, secondary mutations arose that blocked ECA expression (39).
*Escherichia coli*	O-antigen	Inactivating *wzm* and *wzt*, whose gene products transport O-antigen intermediates, disrupted growth, induced morphological defects, and selected for suppressor mutations (45, 46). Inducing O-antigen expression in cells lacking the O-antigen flippases WzxB and WzxE resulted in morphological defects and lysis (47). O-antigen and LPS core mutants produced morphological defects that were reversed by preventing the initiation of O-antigen synthesis or by overexpressing the Und-PP synthase *uppS* or *murA*, which initiates PG synthesis (48).
	ECA	Accumulation of Und-PP-linked ECA intermediates was observed in ECA pathway mutants (41, 43, 49). Inactivating the ECA polymerase *wzyE* was lethal unless combined with mutations that disrupt earlier steps in ECA synthesis (50). ECA pathway mutants produced morphological defects that were reversed by preventing initiation of ECA synthesis or by overexpressing *uppS* or *murA* (51). Inactivating WzxE, which transports ECA intermediates, reduced Und-P levels (52).
	Capsule	Inactivating *kpsM* and *kpsT*, whose gene products transport polysialic acid capsule intermediates, disrupted growth and induced morphological defects (53–55).
*Shigella dysenteriae*	O-antigen	The *S*. *dysenteriae* O-antigen pathway lacking the *wzxB* O-antigen flippase was expressed in *S*. *enterica*, leading to the accumulation of Und-PP-linked O-antigen intermediates (56). Expressing the *S*. *dysenteriae* O-antigen pathway in *E*. *coli* Δ*waaL* cells, which cannot transfer O-antigen from Und-PP to LPS, inhibited growth (57).
*Serratia marcescens*	ECA	ECA pathway mutants produced morphological defects (58).
*Pseudomonas aeruginosa*	O-antigen	Inactivating *wzx*, whose gene product transports O-antigen intermediates, selected for suppressor mutations that prevented O-antigen expression (59).
*Caulobacter crescentus*	O-antigen	Inactivating *wbqL* disrupted O-antigen expression and abolished cell curvature, whereas preventing initiation of O-antigen synthesis restored cell curvature in *wbqL* strains (60).
	Holdfast^*b*^	Inactivating *hfsF* and *hfaD*, whose gene products transport Und-PP-linked holdfast intermediates, gave rise to secondary mutations in genes required for O-antigen synthesis (61).
*Rhizobium meliloti*	Succinoglycan^*c*^	Accumulating Und-PP-linked succinoglycan intermediates was deleterious in late-stage but not in early-stage *exo* pathway mutants (62–64).
*Xanthomonas campestris*	Xanthan^*c*^	Accumulating Und-PP-linked xanthan intermediates was deleterious in late-stage but not in early-stage *gum* pathway mutants (65).
*Myxococcus xanthus*	EPS	Mutations that interrupted EPS synthesis induced shape defects and lysis in sporulating cells (66, 67).
*Bacillus subtilis*	WTA	Mutations that disrupted late steps in WTA synthesis were lethal unless combined with a *tagO* null mutation, which prevents initiation of WTA synthesis (68, 69).
	LTA modification	Overexpressing *csbB* in Δ*yfhO* cells induced morphological defects that were attributed to the accumulation of Und-P-linked intermediates (18).
*Staphylococcus aureus*	WTA	Mutations that disrupted late steps in WTA synthesis were lethal unless combined with a *tarO* null mutation, which prevents initiation of WTA synthesis (70, 71). Antibiotics that block WTA intermediate transport inhibited growth, but this effect was antagonized when combined with tunicamycin, which inhibits the first step in WTA synthesis (72, 73).
*Streptococcus pneumoniae*	Capsule	Mutations that interrupted capsular polysaccharide synthesis selected for isolates containing *cps2E* mutations, which block the first step in capsular polysaccharide synthesis (74, 75).
*Corynebacterium glutamicum*	AG LAM	Inhibiting arabinan synthesis with benzothiazinones (BZTs) was lethal in WT but not in *ubiA* null mutants, which are unable to initiate arabinan synthesis (6). Overexpressing *uppS* increased resistance to BZTs in WT cells (6).
*Mycobacterium tuberculosis*	AG	Inactivating Lcp1, which transfers AG from decaprenyl phosphate to PG, was shown to be lethal (76).

^*a*^ AG, arabinogalactan; ECA, enterobacterial common antigen; EPS, exopolysaccharide; LAM, lipoarabinomannan; LTA, lipoteichoic acid; PG, peptidoglycan; WTA, wall teichoic acid.

^*b*^ Adhesive polysaccharide.

^*c*^ Exopolysaccharide.

## RESOLVING THE UNCERTAINTY BETWEEN SEQUESTRATION AND TOXICITY

The uncertainty over whether Und-P pathway mutant phenotypes resulted from toxic intermediates or sequestration persisted until compelling evidence for sequestration emerged in late 2015 and early 2016. In 2015, Farha et al. demonstrated that supplementing *Staphylococcus aureus* cells with exogenous Und-P suppressed both the inhibitory effect of targocil, which blocks transport of Und-PP-linked WTA intermediates, and lethality due to depletion of TagF, a late-step enzyme in WTA synthesis (77). During this time, Jorgenson et al. also showed that overexpressing either the Und-PP synthase *uppS* (which increases Und-P levels) or the PG transferase *murA* (which increases the competitive advantage of PG synthesis) suppressed morphological, membrane, and motility defects in *E. coli* cells lacking WecE, a late-step glycosyltransferase required for ECA synthesis (51). Similarly, *uppS* and *murA* overexpression suppressed morphological phenotypes in *E. coli* K-12 *wbbL*+ cells lacking WaaC (elongates LPS core) and WaaL (ligates O-antigen to LPS-core) (48). Since pathway defects were reversed in these systems despite continued accumulation of Und-PP-linked intermediates, the implication from these studies was that Und-P pathway-related phenotypes primarily arise from sequestration rather than from toxic intermediates.

Following these seminal reports, additional studies have confirmed that increasing Und-P levels reverses the effects of sequestration (78–80). In particular, *uppS* overexpression reverses the lethal phenotype of a *murJ-amJ* double mutant in *B. subtilis*, demonstrating that Und-P sequestration underlies the essentiality of this gene pair (81). More importantly, since MurJ and AmJ are PG lipid II transporters, this finding points to the existence of additional lipid II transporters in this organism. While sequestration probably explains most Und-P pathway phenotypes, at least one study reported that accumulation of Und-PP-linked colanic acid intermediates, not simply Und-P depletion, was toxic in *E. coli* cells undergoing *de novo* cell wall formation (23). This exception demonstrates that it is not safe to assume that sequestration underlies all Und-P-dependent pathway phenotypes. With the molecular basis for sequestration now established, it is imperative to reevaluate phenotypes (and therefore functions) inferred from any mutations that induce Und-P sequestration, as demonstrated recently (80–82). To that end, Table 2 presents genes that, when disrupted, are expected to sequester Und-P (sequestration associated) in a derivative of *E. coli* K-12.

**TABLE 2:** Glycan synthesis genes linked to Und-P sequestration in *E. coli^a^*

Pathway	Genes	
	Sequestration-associated	Sequestration- independent
Colanic acid	*wzxC*, *wcaD*, *wzc*, *wzb*, and *wza*	*wcaJ*
ECA^*b*^	*rffH*, *rmlA*, *rmlB*, *wecD*, *wecE*, *wecF*, *wecG*, *wzxE*, and *wzyE*	*wecA*
L-Ara4N^*c*^	*arnD*, *arnE*, *arnF*, and *arnT*	*arnC*
Smooth LPS^*d*^	*wbbK*, *wbbJ*, *wbbI*, *wzxB*, *rmlA*, *rmlB*, *rlmC*, *rmlD*, *wbbH*, *waaC*, *waaF*, *waaG*, *waaL*, *waaO*, *waaR*, and *waaU*	*wecA*
O-antigen modification	*gtrA* and *gtrS*	*gtrB*

^*a*^ *E*. *coli* MG1655 *wbbL*+, which expresses O-antigen.

^*b*^ *elyC* null mutations may also induce Und-P sequestration (83, 84).

^*c*^ L-Ara4N, 4-amino-4-deoxy-L-arabinose.

^*d*^ Depletion of LPS transport proteins is also expected to sequester Und-P in ECA, O-antigen, and colanic acid intermediates.

## MANIPULATING UND-P SEQUESTRATION HELPS RECONSTITUTE UND-P PATHWAYS *IN VITRO*

Beyond its role as a cellular stressor, Und-P sequestration has been exploited to advance our understanding of Und-P-utilizing pathways. For example, in 2021, *uppS* overexpression was used in *E. coli* ECA and colanic acid pathway mutants to generate large quantities of Und-PP-linked intermediates (52, 85). Having sufficient quantities of these intermediates in the membrane helped validate the *in vitro* reconstruction of the ECA and colanic acid pathways by providing standards for comparison. Although Und-P sequestration is typically deleterious, *uppS* overexpression helped ensure sufficient PG precursor formation in these sequestration derivatives by increasing Und-P availability, which generally increases synthesis of all Und-P-dependent polymers (19). Trapping Und-P in colanic acid intermediates also helped determine the functional roles of the WcaK and WcaL transferases, which catalyze the last two steps of colanic acid biosynthesis (85). In summary, Und-P sequestration has been used to better understand biosynthetic pathways and may also prove useful in applications where chemical synthesis of Und-P(P)-linked intermediates is challenging.

## CHEMICALLY INDUCING UND-P SEQUESTRATION IDENTIFIES UND-P TRANSPORTERS

More recently, Und-P sequestration has been leveraged to identify the long-sought-after Und-P translocases. Once Und-PP is dephosphorylated by BacA and PAP2 family proteins, Und-P must return to the cytoplasmic leaflet of the inner (cell) membrane to initiate another round of assembly. While structural studies indicate that BacA may participate in Und-P flipping (86, 87), the exact mechanism of Und-P transport has remained elusive. Significant progress toward understanding Und-P flipping came in 2022 when two independent studies identified putative Und-P translocases by using the Und-P sequestering antibiotic amphomycin and its analog MX2401 (see Table 3 for a list of Und-P sequestering antibiotics).

**TABLE 3:** Antibiotics that directly sequester Und-P(P) or Und-P(P)-linked intermediates

Mechanism of action	Antibiotic
Und-P sequestration	Amphomycin (88), aspartocin D (89), cilagicin (90), friulimicin (91), laspartomycin C (92), and MX2401 (93)
Und-PP sequestration	Bacitracin (94) and cilagicin (90)
DP^*a*^ sequestration	BTZ043 (6), OPC-167832 (95), and PBTZ169 (96)
Lipid II^PG^ sequestration	CDFI (97), DMPI (97), enduracidin (98), HNP1 (99), Lys^M^ (100), lysobactin (101), malacidin (102), mannopeptimycin (103), mersacidin (104), mutacin (105), nisin (106), plectasin (107), ramoplanin (98), texicobactin (108), and vancomycin (101)
Lipid II^WTA^ sequestration	Lysobactin (101), targocil (109), and targocil-II (110)

^*a*^ Decaprenyl phosphate (C_50_-P).

In the first study, Roney and Rudner screened for genes whose overexpression confers increased resistance to MX2401 in *B. subtilis*, as well as mutations that increase sensitivity to amphomycin in *S. aureus* (12). Meanwhile, Sit et al. used amphomycin to investigate genes linked with Und-P recycling in *Vibrio cholerae* and *S. aureus* (13). In both cases, DedA family proteins and DUF368 domain-containing proteins were implicated in Und-P recycling. Subsequent analyses using fluorescently labeled MX2401 and amphomycin revealed that Und-P accumulates on the outer leaflet of the cell membrane in cells lacking DedA and DUF368 proteins, strongly supporting their role in Und-P flipping (12, 13). Since deleting the genes encoding DedA and DUF368 proteins indirectly sequesters Und-P, it follows that cells lacking these proteins exhibit phenotypes similar to those observed for Und-P sequestration (12, 13, 111–113). Amphomycin was later used to probe the interaction between Und-P and UptA, a DedA family protein (89). In summary, chemically inducing Und-P sequestration helped clarify how Und-P is cycled through the membrane (33).

## UNDERSTANDING THE EFFECTS OF UND-P SEQUESTRATION HELPS REVEAL THE MECHANISM OF ACTION OF ANTIBIOTICS

Another recent study showed how understanding the effects of Und-P sequestration revealed an antibiotic’s mechanism of action (MOA). In 2022, Wang et al. screened for biosynthetic gene clusters (BGCs) predicted to encode novel lipopeptide antibiotics (90). One of their findings included a BGC harboring three non-ribosomal synthetase open reading frames, *cilCDE*, from the gram-positive bacterium *Paenibacillus mucilaginosus*. The authors synthesized the product from the *cil* BGC, a lipopeptide (termed cilagicin) with potent antibacterial activity against gram-positive bacteria, including *S. aureus*, *Enterococci* pathogens, and *Clostridioides difficile*. To determine the MOA of cilagicin, the authors attempted to isolate cilagicin-resistant cells, but these efforts were unsuccessful. Interestingly, liquid chromatography-mass spectrometry analysis revealed the accumulation of the PG lipid II precursor UDP-*N*-acetylmuramic acid pentapeptide (which is attached to Und-P by MraY) in cilagicin-treated cells, suggesting that cilagicin sequesters Und-P. Suspecting this, the authors tested whether supplementing cells with Und-P and Und-PP could reduce cilagicin’s bactericidal activity against *S. aureus* cells, which they found to be the case. Follow-up analyses using isothermal titration calorimetry revealed that cilagicin binds Und-P and Und-PP. In total, these findings demonstrate how a deep understanding of Und-P sequestration was key to uncovering the MOA of a previously hidden antibiotic.

## INTRINSIC CONTROLS ON UND-P SEQUESTRATION

Given that Und-P sequestration is deleterious, a key question is whether bacteria encode factors to counteract this phenomenon. This question has been partially addressed in both gram-positive and gram-negative bacteria. In 2024, Roney and Rudner discovered that the alternative sigma factor SigM is activated in *B. subtilis* when Und-P is sequestered, either in response to antibiotics that bind to Und-PP-linked intermediates (ramoplanin and vancomycin) or by depletion of proteins (MurG and MurJ) that bind and transport Und-PP-linked intermediates (114). Since SigM regulates genes involved in cell wall synthesis (115), SigM activation likely increases the competitive advantage of PG synthesis in this system. This would explain why *B. subtilis* cells lacking SigM grow poorly when Und-P becomes limiting (114). In a follow-up study, the same authors showed that SigM activation increases the expression of two enzymes, UshA and UpsH, that are predicted to cleave Und-P from non-essential polymers in *B. subtilis* (116). These findings indicate that bacteria like *B. subtilis* employ controls to prioritize PG synthesis when Und-P becomes limiting.

Separately, in *P. aeruginosa*, Marmont et al. isolated a hyperactive variant of the phosphoglycosyl transferase MraY, which transfers PG precursors to Und-P. This variant (MraY[T23P]) rescued the growth of a Δ*ponB*Δ*lpoA* mutant, which is defective in PG synthesis, on minimal medium (117). Follow-up experiments revealed that cells expressing MraY(T23P) accumulate PG lipid II, an indication that MraY activity is feedback inhibited by excessive amounts (i.e., sequestration) of this intermediate. Notably, MraY(T23P)-expressing cells were observed to produce less O-antigen, a glycoconjugate required to protect against self-killing by R2-pyocin (118). Thus, *P. aeruginosa* employs a feedback control mechanism to prevent sequestration of PG intermediates, likely as a fail-safe against self-killing. Overproducing *E. coli* MraY(T23P) also rescued an *E. coli* mutant defective for PG synthesis, indicating the MraY regulatory mechanism is conserved. A key question is whether feedback inhibition by Und-P(P)-linked intermediates is a common feature of PGTs or if this particular mechanism evolved to be pathway specific.

Finally, recent studies of the gram-negative bacterium *Coxiella burnetii* have uncovered another potential Und-P regulatory mechanism. When cultured *in vitro*, *Coxiella* undergoes LPS phase variation, where it transitions from expressing full-length phase I LPS to expressing a shortened version termed phase II that lacks outer core sugars and O-antigen (119). Recently, whole-genome sequencing has revealed that strains expressing phase II LPS harbor inactivating mutations in *cbu0533* (120), whose gene product is expected to initiate O-antigen synthesis. Interestingly, *cbu0533* encodes a stretch of five leucine residues that converts to four in phase II mutants and re-expands to five (which restores phase I LPS expression) upon passaging in certain media and animal models (121). Thus, *Coxiella* appears to have evolved a genetic toggle that modulates O-antigen initiation, presumably to maintain a steady supply of Und-P. Note that preventing O-antigen initiation is expected to increase the amount of Und-P available for PG synthesis and other Und-P-dependent pathways.

In summary, these findings demonstrate that bacteria can regulate the flow of Und-P in order to avoid or reverse the effects of Und-P sequestration. Future studies are needed to determine whether additional controls exist and, if so, whether they can be exploited for therapeutic purposes.

## CONCLUDING THOUGHTS

The bacterial cell envelope is coated with glycans that protect against antibiotics, immune systems, and environmental hazards. To assemble these glycans, bacteria employ a reusable carrier lipid known as Und-P. When Und-P becomes trapped in dead-end intermediates, a situation known as sequestration, it cannot be recycled for reuse. Und-P sequestration reduces the amount of Und-P available for PG synthesis, which is essential for viability, as well as any other Und-P-dependent pathway within the cell. For decades, the precise mechanism of Und-P sequestration remained unclear until recent advances firmly established its molecular basis. By unraveling the mechanism of Und-P sequestration, we have significantly clarified our understanding of gene essentiality, glycan function, antibiotic action, and envelope assembly. Since Und-P pathways are ubiquitous, the effects of Und-P sequestration are likely to manifest in increasingly unique ways as more bacterial systems are investigated. Thus, continued attention to the mechanism of Und-P sequestration is essential, not only to interpret phenotypes accurately but also to further illuminate hidden aspects of envelope biogenesis and guide the development of new strategies to disrupt the cell envelope.

## References

[1] Silhavy TJ, Kahne D, Walker S. 2010. The bacterial cell envelope. Cold Spring Harb Perspect Biol 2:a000414. doi:10.1101/cshperspect.a000414 20452953 PMC2857177

[2] Tytgat HLP, Lebeer S. 2014. The sweet tooth of bacteria: common themes in bacterial glycoconjugates. Microbiol Mol Biol Rev 78:372–417. doi:10.1128/MMBR.00007-14 25184559 PMC4187687

[3] Garde S, Chodisetti PK, Reddy M. 2021. Peptidoglycan: structure, synthesis, and regulation. EcoSal Plus 9:eESP-0010-2020. doi:10.1128/ecosalplus.ESP-0010-2020 PMC11168573 33470191

[4] Manat G, Roure S, Auger R, Bouhss A, Barreteau H, Mengin-Lecreulx D, Touzé T. 2014. Deciphering the metabolism of undecaprenyl-phosphate: the bacterial cell-wall unit carrier at the membrane frontier. Microb Drug Resist 20:199–214. doi:10.1089/mdr.2014.0035 24799078 PMC4050452

[5] Kaur D, Brennan PJ, Crick DC. 2004. Decaprenyl diphosphate synthesis in Mycobacterium tuberculosis. J Bacteriol 186:7564–7570. doi:10.1128/JB.186.22.7564-7570.2004 15516568 PMC524883

[6] Grover S, Alderwick LJ, Mishra AK, Krumbach K, Marienhagen J, Eggeling L, Bhatt A, Besra GS. 2014. Benzothiazinones mediate killing of Corynebacterineae by blocking decaprenyl phosphate recycling involved in cell wall biosynthesis. J Biol Chem 289:6177–6187. doi:10.1074/jbc.M113.522623 24446451 PMC3937683

[7] Ishii K, Sagami H, Ogura K. 1986. A novel prenyltransferase from Paracoccus denitrificans. Biochem J 233:773–777. doi:10.1042/bj2330773 3707524 PMC1153098

[8] El Ghachi M, Bouhss A, Blanot D, Mengin-Lecreulx D. 2004. The bacA gene of Escherichia coli encodes an undecaprenyl pyrophosphate phosphatase activity. J Biol Chem 279:30106–30113. doi:10.1074/jbc.M401701200 15138271

[9] El Ghachi M, Derbise A, Bouhss A, Mengin-Lecreulx D. 2005. Identification of multiple genes encoding membrane proteins with undecaprenyl pyrophosphate phosphatase (UppP) activity in Escherichia coli. J Biol Chem 280:18689–18695. doi:10.1074/jbc.M412277200 15778224

[10] Bernard R, El Ghachi M, Mengin-Lecreulx D, Chippaux M, Denizot F. 2005. BcrC from Bacillus subtilis acts as an undecaprenyl pyrophosphate phosphatase in bacitracin resistance. J Biol Chem 280:28852–28857. doi:10.1074/jbc.M413750200 15946938

[11] Workman SD, Strynadka NCJ. 2020. A slippery scaffold: synthesis and recycling of the bacterial cell wall carrier lipid. J Mol Biol 432:4964–4982. doi:10.1016/j.jmb.2020.03.025 32234311

[12] Roney IJ, Rudner DZ. 2023. Two broadly conserved families of polyprenyl-phosphate transporters. Nature 613:729–734. doi:10.1038/s41586-022-05587-z 36450357 PMC10184681

[13] Sit B, Srisuknimit V, Bueno E, Zingl FG, Hullahalli K, Cava F, Waldor MK. 2023. Undecaprenyl phosphate translocases confer conditional microbial fitness. Nature 613:721–728. doi:10.1038/s41586-022-05569-1 36450355 PMC9876793

[14] Tatar LD, Marolda CL, Polischuk AN, van Leeuwen D, Valvano MA. 2007. An Escherichia coli undecaprenyl-pyrophosphate phosphatase implicated in undecaprenyl phosphate recycling. Microbiology (Reading) 153:2518–2529. doi:10.1099/mic.0.2007/006312-0 17660416

[15] Manat G, El Ghachi M, Auger R, Baouche K, Olatunji S, Kerff F, Touzé T, Mengin-Lecreulx D, Bouhss A. 2015. Membrane topology and biochemical characterization of the Escherichia coli BacA undecaprenyl-pyrophosphate phosphatase. PLoS One 10:e0142870. doi:10.1371/journal.pone.0142870 26560897 PMC4641660

[16] Hartley MD, Imperiali B. 2012. At the membrane frontier: a prospectus on the remarkable evolutionary conservation of polyprenols and polyprenyl-phosphates. Arch Biochem Biophys 517:83–97. doi:10.1016/j.abb.2011.10.018 22093697 PMC3253937

[17] Raetz CRH, Reynolds CM, Trent MS, Bishop RE. 2007. Lipid A modification systems in Gram-negative bacteria. Annu Rev Biochem 76:295–329. doi:10.1146/annurev.biochem.76.010307.145803 17362200 PMC2569861

[18] Inoue H, Suzuki D, Asai K. 2013. A putative bactoprenol glycosyltransferase, CsbB, in Bacillus subtilis activates SigM in the absence of co-transcribed YfhO. Biochem Biophys Res Commun 436:6–11. doi:10.1016/j.bbrc.2013.04.064 23632331

[19] Kay EJ, Dooda MK, Bryant JC, Reid AJ, Wren BW, Troutman JM, Jorgenson MA. 2024. Engineering Escherichia coli for increased Und-P availability leads to material improvements in glycan expression technology. Microb Cell Fact 23:72. doi:10.1186/s12934-024-02339-8 38429691 PMC10908060

[20] Barreteau H, Magnet S, El Ghachi M, Touzé T, Arthur M, Mengin-Lecreulx D, Blanot D. 2009. Quantitative high-performance liquid chromatography analysis of the pool levels of undecaprenyl phosphate and its derivatives in bacterial membranes. J Chromatogr B Analyt Technol Biomed Life Sci 877:213–220. doi:10.1016/j.jchromb.2008.12.010 19110475

[21] Rai AK, Mitchell AM. 2020. Enterobacterial common antigen: synthesis and function of an enigmatic molecule. mBio 11. doi:10.1128/mBio.01914-20 PMC7439462 32788387

[22] Bertani B, Ruiz N. 2018. Function and biogenesis of lipopolysaccharides. EcoSal Plus 8. doi:10.1128/ecosalplus.ESP-0001-2018 PMC6091223 30066669

[23] Ranjit DK, Young KD. 2016. Colanic acid intermediates prevent de novo shape recovery of Escherichia coli spheroplasts, calling into question biological roles previously attributed to colanic acid. J Bacteriol 198:1230–1240. doi:10.1128/JB.01034-15 26833417 PMC4859594

[24] Weissborn AC, Rumley MK, Kennedy EP. 1991. Biosynthesis of membrane-derived oligosaccharides. Membrane-bound glucosyltransferase system from Escherichia coli requires polyprenyl phosphate. J Biol Chem 266:8062–8067. doi:10.1016/S0021-9258(18)92940-6 1827116

[25] Allison GE, Verma NK. 2000. Serotype-converting bacteriophages and O-antigen modification in Shigella flexneri. Trends Microbiol 8:17–23. doi:10.1016/s0966-842x(99)01646-7 10637639

[26] Liu D, Reeves PR. 1994. Escherichia coli K12 regains its O antigen. Microbiology (Reading, Engl) 140:49–57. doi:10.1099/13500872-140-1-49 7512872

[27] Jeong H, Barbe V, Lee CH, Vallenet D, Yu DS, Choi S-H, Couloux A, Lee S-W, Yoon SH, Cattolico L, Hur C-G, Park H-S, Ségurens B, Kim SC, Oh TK, Lenski RE, Studier FW, Daegelen P, Kim JF. 2009. Genome sequences of Escherichia coli B strains REL606 and BL21(DE3). J Mol Biol 394:644–652. doi:10.1016/j.jmb.2009.09.052 19786035

[28] Rajagopal M, Walker S. 2017. Envelope structures of Gram-positive bacteria. Curr Top Microbiol Immunol 404:1–44. doi:10.1007/82_2015_5021 26919863 PMC5002265

[29] Arbour CA, Nagar R, Bernstein HM, Ghosh S, Al-Sammarraie Y, Dorfmueller HC, Ferguson MAJ, Stanley-Wall NR, Imperiali B. 2023. Defining early steps in Bacillus subtilis biofilm biosynthesis. mBio 14:e0094823. doi:10.1128/mbio.00948-23 37650625 PMC10653937

[30] Rismondo J, Percy MG, Gründling A. 2018. Discovery of genes required for lipoteichoic acid glycosylation predicts two distinct mechanisms for wall teichoic acid glycosylation. J Biol Chem 293:3293–3306. doi:10.1074/jbc.RA117.001614 29343515 PMC5836110

[31] Rismondo J, Gillis A, Gründling A. 2021. Modifications of cell wall polymers in Gram-positive bacteria by multi-component transmembrane glycosylation systems. Curr Opin Microbiol 60:24–33. doi:10.1016/j.mib.2021.01.007 33578058 PMC8035078

[32] Zhao H, Sun Y, Peters JM, Gross CA, Garner EC, Helmann JD. 2016. Depletion of undecaprenyl pyrophosphate phosphatases disrupts cell envelope biogenesis in Bacillus subtilis. J Bacteriol 198:2925–2935. doi:10.1128/JB.00507-16 27528508 PMC5055597

[33] Jorgenson MA, Bryant JC. 2021. A genetic screen to identify factors affected by undecaprenyl phosphate recycling uncovers novel connections to morphogenesis in Escherichia coli. Mol Microbiol 115:191–207. doi:10.1111/mmi.14609 32979869 PMC10568968

[34] Yuasa R, Levinthal M, Nikaido H. 1969. Biosynthesis of cell wall lipopolysaccharide in mutants of Salmonella. V. A mutant of Salmonella typhimurium defective in the synthesis of cytidine diphosphoabequose. J Bacteriol 100:433–444. doi:10.1128/jb.100.1.433-444.1969 4899003 PMC315411

[35] Wright A, Dankert M, Fennessey P, Robbins PW. 1967. Characterization of a polyisoprenoid compound functional in O-antigen biosynthesis. Proc Natl Acad Sci USA 57:1798–1803. doi:10.1073/pnas.57.6.1798 4291948 PMC224549

[36] Watkinson RJ, Hussey H, Baddiley J. 1971. Shared lipid phosphate carrier in the biosynthesis of teichoic acid and peptidoglycan. Nat New Biol 229:57–59. doi:10.1038/newbio229057a0 4250444

[37] Anderson RG, Hussey H, Baddiley J. 1972. The mechanism of wall synthesis in bacteria. The organization of enzymes and isoprenoid phosphates in the membrane. Biochem J 127:11–25. doi:10.1042/bj1270011 4627447 PMC1178555

[38] Masson L, Holbein BE. 1985. Role of lipid intermediate(s) in the synthesis of serogroup B Neisseria meningitidis capsular polysaccharide. J Bacteriol 161:861–867. doi:10.1128/jb.161.3.861-867.1985 3918990 PMC214976

[39] Mäkelä PH, Schmidt G, Mayer H, Nikaido H, Whang HY, Neter E. 1976. Enterobacterial common antigen in rfb deletion mutants of Salmonella typhimurium. J Bacteriol 127:1141–1149. doi:10.1128/jb.127.3.1141-1149.1976 783131 PMC232905

[40] Rick PD, Mayer H, Neumeyer BA, Wolski S, Bitter-Suermann D. 1985. Biosynthesis of enterobacterial common antigen. J Bacteriol 162:494–503. doi:10.1128/jb.162.2.494-503.1985 3886625 PMC218875

[41] Rick PD, Wolski S, Barr K, Ward S, Ramsay-Sharer L. 1988. Accumulation of a lipid-linked intermediate involved in enterobacterial common antigen synthesis in Salmonella typhimurium mutants lacking dTDP-glucose pyrophosphorylase. J Bacteriol 170:4008–4014. doi:10.1128/jb.170.9.4008-4014.1988 2842298 PMC211403

[42] Cain BD, Norton PJ, Eubanks W, Nick HS, Allen CM. 1993. Amplification of the bacA gene confers bacitracin resistance to Escherichia coli. J Bacteriol 175:3784–3789. doi:10.1128/jb.175.12.3784-3789.1993 8389741 PMC204795

[43] Danese PN, Oliver GR, Barr K, Bowman GD, Rick PD, Silhavy TJ. 1998. Accumulation of the enterobacterial common antigen lipid II biosynthetic intermediate stimulates degP transcription in Escherichia coli. J Bacteriol 180:5875–5884. doi:10.1128/JB.180.22.5875-5884.1998 9811644 PMC107660

[44] Liu MA, Stent TL, Hong YQ, Reeves PR. 2015. Inefficient translocation of a truncated O unit by a Salmonella Wzx affects both O-antigen production and cell growth. FEMS Microbiol Lett 362:fnv053. doi:10.1093/femsle/fnv053 25837817

[45] Clarke BR, Cuthbertson L, Whitfield C. 2004. Nonreducing terminal modifications determine the chain length of polymannose O antigens of Escherichia coli and couple chain termination to polymer export via an ATP-binding cassette transporter. J Biol Chem 279:35709–35718. doi:10.1074/jbc.M404738200 15184370

[46] Cuthbertson L, Powers J, Whitfield C. 2005. The C-terminal domain of the nucleotide-binding domain protein Wzt determines substrate specificity in the ATP-binding cassette transporter for the lipopolysaccharide O-antigens in Escherichia coli serotypes O8 and O9a. J Biol Chem 280:30310–30319. doi:10.1074/jbc.M504371200 15980069

[47] Marolda CL, Tatar LD, Alaimo C, Aebi M, Valvano MA. 2006. Interplay of the Wzx translocase and the corresponding polymerase and chain length regulator proteins in the translocation and periplasmic assembly of lipopolysaccharide o antigen. J Bacteriol 188:5124–5135. doi:10.1128/JB.00461-06 16816184 PMC1539953

[48] Jorgenson MA, Young KD. 2016. Interrupting biosynthesis of O antigen or the lipopolysaccharide core produces morphological defects in Escherichia coli by sequestering undecaprenyl phosphate. J Bacteriol 198:3070–3079. doi:10.1128/JB.00550-16 27573014 PMC5075036

[49] Meier-Dieter U, Starman R, Barr K, Mayer H, Rick PD. 1990. Biosynthesis of enterobacterial common antigen in Escherichia coli. Biochemical characterization of Tn10 insertion mutants defective in enterobacterial common antigen synthesis. J Biol Chem 265:13490–13497. doi:10.1016/S0021-9258(18)77373-0 2166030

[50] Kajimura J, Rahman A, Rick PD. 2005. Assembly of cyclic enterobacterial common antigen in Escherichia coli K-12. J Bacteriol 187:6917–6927. doi:10.1128/JB.187.20.6917-6927.2005 16199561 PMC1251615

[51] Jorgenson MA, Kannan S, Laubacher ME, Young KD. 2016. Dead-end intermediates in the enterobacterial common antigen pathway induce morphological defects in Escherichia coli by competing for undecaprenyl phosphate. Mol Microbiol 100:1–14. doi:10.1111/mmi.13284 26593043 PMC4845916

[52] Eade CR, Wallen TW, Gates CE, Oliverio CL, Scarbrough BA, Reid AJ, Jorgenson MA, Young KD, Troutman JM. 2021. Making the enterobacterial common antigen glycan and measuring its substrate sequestration. ACS Chem Biol 16:691–700. doi:10.1021/acschembio.0c00983 33740380 PMC8080848

[53] Silver RP, Vann WF, Aaronson W. 1984. Genetic and molecular analyses of Escherichia coli K1 antigen genes. J Bacteriol 157:568–575. doi:10.1128/jb.157.2.568-575.1984 6319367 PMC215284

[54] Pavelka MS, Hayes SF, Silver RP. 1994. Characterization of KpsT, the ATP-binding component of the ABC-transporter involved with the export of capsular polysialic acid in Escherichia coli K1. J Biol Chem 269:20149–20158. doi:10.1016/S0021-9258(17)32139-7 8051103

[55] Bliss JM, Garon CF, Silver RP. 1996. Polysialic acid export in Escherichia coli K1: the role of KpsT, the ATP-binding component of an ABC transporter, in chain translocation. Glycobiology 6:445–452. doi:10.1093/glycob/6.4.445 8842709

[56] Liu D, Cole RA, Reeves PR. 1996. An O-antigen processing function for Wzx (RfbX): a promising candidate for O-unit flippase. J Bacteriol 178:2102–2107. doi:10.1128/jb.178.7.2102-2107.1996 8606190 PMC177911

[57] Klena JD, Schnaitman CA. 1993. Function of the rfb gene cluster and the rfe gene in the synthesis of O antigen by Shigella dysenteriae 1. Mol Microbiol 9:393–402. doi:10.1111/j.1365-2958.1993.tb01700.x 7692219

[58] Castelli ME, Fedrigo GV, Clementín AL, Ielmini MV, Feldman MF, García Véscovi E. 2008. Enterobacterial common antigen integrity is a checkpoint for flagellar biogenesis in Serratia marcescens. J Bacteriol 190:213–220. doi:10.1128/JB.01348-07 17981971 PMC2223741

[59] Burrows LL, Lam JS. 1999. Effect of wzx (rfbX) mutations on A-band and B-band lipopolysaccharide biosynthesis in Pseudomonas aeruginosa O5. J Bacteriol 181:973–980. doi:10.1128/JB.181.3.973-980.1999 9922263 PMC93466

[60] Cabeen MT, Murolo MA, Briegel A, Bui NK, Vollmer W, Ausmees N, Jensen GJ, Jacobs-Wagner C. 2010. Mutations in the lipopolysaccharide biosynthesis pathway interfere with crescentin-mediated cell curvature in Caulobacter crescentus. J Bacteriol 192:3368–3378. doi:10.1128/JB.01371-09 20435724 PMC2897673

[61] Hardy GG, Toh E, Berne C, Brun YV. 2018. Mutations in sugar-nucleotide synthesis genes restore holdfast polysaccharide anchoring to Caulobacter crescentus holdfast anchor mutants. J Bacteriol 200:e00597-17. doi:10.1128/JB.00597-17 29158242 PMC5763047

[62] Reuber TL, Long S, Walker GC. 1991. Regulation of Rhizobium meliloti exo genes in free-living cells and in planta examined by using TnphoA fusions. J Bacteriol 173:426–434. doi:10.1128/jb.173.2.426-434.1991 1846141 PMC207029

[63] Reuber TL, Walker GC. 1993. Biosynthesis of succinoglycan, a symbiotically important exopolysaccharide of Rhizobium meliloti. Cell 74:269–280. doi:10.1016/0092-8674(93)90418-p 8343955

[64] Glucksmann MA, Reuber TL, Walker GC. 1993. Genes needed for the modification, polymerization, export, and processing of succinoglycan by Rhizobium meliloti: a model for succinoglycan biosynthesis. J Bacteriol 175:7045–7055. doi:10.1128/jb.175.21.7045-7055.1993 8226646 PMC206832

[65] Katzen F, Ferreiro DU, Oddo CG, Ielmini MV, Becker A, Pühler A, Ielpi L. 1998. Xanthomonas campestris pv. campestris gum mutants: effects on xanthan biosynthesis and plant virulence. J Bacteriol 180:1607–1617. doi:10.1128/JB.180.7.1607-1617.1998 9537354 PMC107069

[66] Holkenbrink C, Hoiczyk E, Kahnt J, Higgs PI. 2014. Synthesis and assembly of a novel glycan layer in Myxococcus xanthus spores. J Biol Chem 289:32364–32378. doi:10.1074/jbc.M114.595504 25271164 PMC4231708

[67] Pérez-Burgos M, García-Romero I, Valvano MA, Søgaard Andersen L. 2020. Identification of the Wzx flippase, Wzy polymerase and sugar-modifying enzymes for spore coat polysaccharide biosynthesis in Myxococcus xanthus. Mol Microbiol 113:1189–1208. doi:10.1111/mmi.14486 32064693

[68] Bhavsar AP, Beveridge TJ, Brown ED. 2001. Precise deletion of tagD and controlled depletion of its product, glycerol 3-phosphate cytidylyltransferase, leads to irregular morphology and lysis of Bacillus subtilis grown at physiological temperature. J Bacteriol 183:6688–6693. doi:10.1128/JB.183.22.6688-6693.2001 11673441 PMC95502

[69] D’Elia MA, Millar KE, Beveridge TJ, Brown ED. 2006. Wall teichoic acid polymers are dispensable for cell viability in Bacillus subtilis. J Bacteriol 188:8313–8316. doi:10.1128/JB.01336-06 17012386 PMC1698200

[70] D’Elia MA, Pereira MP, Chung YS, Zhao W, Chau A, Kenney TJ, Sulavik MC, Black TA, Brown ED. 2006. Lesions in teichoic acid biosynthesis in Staphylococcus aureus lead to a lethal gain of function in the otherwise dispensable pathway. J Bacteriol 188:4183–4189. doi:10.1128/JB.00197-06 16740924 PMC1482942

[71] Chan YGY, Frankel MB, Dengler V, Schneewind O, Missiakas D. 2013. Staphylococcus aureus mutants lacking the LytR-CpsA-Psr family of enzymes release cell wall teichoic acids into the extracellular medium. J Bacteriol 195:4650–4659. doi:10.1128/JB.00544-13 23935043 PMC3807444

[72] Swoboda JG, Meredith TC, Campbell J, Brown S, Suzuki T, Bollenbach T, Malhowski AJ, Kishony R, Gilmore MS, Walker S. 2009. Discovery of a small molecule that blocks wall teichoic acid biosynthesis in Staphylococcus aureus. ACS Chem Biol 4:875–883. doi:10.1021/cb900151k 19689117 PMC2787957

[73] Campbell J, Singh AK, Swoboda JG, Gilmore MS, Wilkinson BJ, Walker S. 2012. An antibiotic that inhibits a late step in wall teichoic acid biosynthesis induces the cell wall stress stimulon in Staphylococcus aureus. Antimicrob Agents Chemother 56:1810–1820. doi:10.1128/AAC.05938-11 22290958 PMC3318382

[74] Xayarath B, Yother J. 2007. Mutations blocking side chain assembly, polymerization, or transport of a Wzy-dependent Streptococcus pneumoniae capsule are lethal in the absence of suppressor mutations and can affect polymer transfer to the cell wall. J Bacteriol 189:3369–3381. doi:10.1128/JB.01938-06 17322316 PMC1855910

[75] James DBA, Yother J. 2012. Genetic and biochemical characterizations of enzymes involved in Streptococcus pneumoniae serotype 2 capsule synthesis demonstrate that Cps2T (WchF) catalyzes the committed step by addition of β1-4 rhamnose, the second sugar residue in the repeat unit. J Bacteriol 194:6479–6489. doi:10.1128/JB.01135-12 23002227 PMC3497468

[76] Harrison J, Lloyd G, Joe M, Lowary TL, Reynolds E, Walters-Morgan H, Bhatt A, Lovering A, Besra GS, Alderwick LJ. 2016. Lcp1 Is a phosphotransferase responsible for ligating arabinogalactan to peptidoglycan in Mycobacterium tuberculosis. mBio 7:e00972-16. doi:10.1128/mBio.00972-16 27486192 PMC4981717

[77] Farha MA, Czarny TL, Myers CL, Worrall LJ, French S, Conrady DG, Wang Y, Oldfield E, Strynadka NCJ, Brown ED. 2015. Antagonism screen for inhibitors of bacterial cell wall biogenesis uncovers an inhibitor of undecaprenyl diphosphate synthase. Proc Natl Acad Sci USA 112:11048–11053. doi:10.1073/pnas.1511751112 26283394 PMC4568241

[78] Maczuga N, Tran ENH, Qin J, Morona R. 2022. Interdependence of Shigella flexneri O antigen and enterobacterial common antigen biosynthetic pathways. J Bacteriol 204:e0054621. doi:10.1128/jb.00546-21 35293778 PMC9017295

[79] Qin J, Hong Y, Maczuga NT, Morona R, Totsika M. 2025. Tolerance mechanisms in polysaccharide biosynthesis: implications for undecaprenol phosphate recycling in Escherichia coli and Shigella flexneri. PLoS Genet 21:e1011591. doi:10.1371/journal.pgen.1011591 39883743 PMC11813082

[80] Jebeli L, McDaniels TA, Ho DTT, Tahir H, Kai-Ming NL, Mcgaw M, Karlic KI, Lewis JM, Scott NE. 2025. The late-stage steps of Burkholderia cenocepacia protein O-linked glycan biosynthesis are conditionally essential. J Biol Chem 301:108515. doi:10.1016/j.jbc.2025.108515 40286851 PMC12152626

[81] Englehart K, Dworkin J. 2025. Bacillus subtilis MurJ and Amj Lipid II flippases are not essential for growth. J Bacteriol 207:e0007825. doi:10.1128/jb.00078-25 40183557 PMC12096821

[82] Tan YH, Chen Y, Chu WHW, Sham LT, Gan YH. 2020. Cell envelope defects of different capsule-null mutants in K1 hypervirulent Klebsiella pneumoniae can affect bacterial pathogenesis. Mol Microbiol 113:889–905. doi:10.1111/mmi.14447 31912541 PMC7317392

[83] Paradis-Bleau C, Kritikos G, Orlova K, Typas A, Bernhardt TG. 2014. A genome-wide screen for bacterial envelope biogenesis mutants identifies a novel factor involved in cell wall precursor metabolism. PLoS Genet 10:e1004056. doi:10.1371/journal.pgen.1004056 24391520 PMC3879167

[84] Rai AK, Carr JF, Bautista DE, Wang W, Mitchell AM. 2021. ElyC and cyclic enterobacterial common antigen regulate synthesis of phosphoglyceride-linked enterobacterial common antigen. mBio 12:e0284621. doi:10.1128/mBio.02846-21 34809459 PMC8609368

[85] Reid AJ, Eade CR, Jones KJ, Jorgenson MA, Troutman JM. 2021. Tracking colanic acid repeat unit formation from stepwise biosynthesis inactivation in Escherichia coli. Biochemistry 60:2221–2230. doi:10.1021/acs.biochem.1c00314 34159784 PMC8277737

[86] El Ghachi M, Howe N, Huang C-Y, Olieric V, Warshamanage R, Touzé T, Weichert D, Stansfeld PJ, Wang M, Kerff F, Caffrey M. 2018. Crystal structure of undecaprenyl-pyrophosphate phosphatase and its role in peptidoglycan biosynthesis. Nat Commun 9:1078. doi:10.1038/s41467-018-03477-5 29540682 PMC5852022

[87] Workman SD, Worrall LJ, Strynadka NCJ. 2018. Crystal structure of an intramembranal phosphatase central to bacterial cell-wall peptidoglycan biosynthesis and lipid recycling. Nat Commun 9:1159. doi:10.1038/s41467-018-03547-8 29559664 PMC5861054

[88] Singh M, Chang J, Coffman L, Kim SJ. 2016. Solid-state NMR characterization of amphomycin effects on peptidoglycan and wall teichoic acid biosyntheses in Staphylococcus aureus. Sci Rep 6:31757. doi:10.1038/srep31757 27538449 PMC4990924

[89] Oluwole AO, Kalmankar NV, Guida M, Bennett JL, Poce G, Bolla JR, Robinson CV. 2024. Lipopeptide antibiotics disrupt interactions of undecaprenyl phosphate with UptA. Proc Natl Acad Sci USA 121:e2408315121. doi:10.1073/pnas.2408315121 39361645 PMC11474028

[90] Wang Z, Koirala B, Hernandez Y, Zimmerman M, Brady SF. 2022. Bioinformatic prospecting and synthesis of a bifunctional lipopeptide antibiotic that evades resistance. Science 376:991–996. doi:10.1126/science.abn4213 35617397 PMC10904332

[91] Schneider T, Gries K, Josten M, Wiedemann I, Pelzer S, Labischinski H, Sahl HG. 2009. The lipopeptide antibiotic Friulimicin B inhibits cell wall biosynthesis through complex formation with bactoprenol phosphate. Antimicrob Agents Chemother 53:1610–1618. doi:10.1128/AAC.01040-08 19164139 PMC2663061

[92] Kleijn LHJ, Oppedijk SF, ’t Hart P, van Harten RM, Martin-Visscher LA, Kemmink J, Breukink E, Martin NI. 2016. Total synthesis of laspartomycin C and characterization of its antibacterial mechanism of action. J Med Chem 59:3569–3574. doi:10.1021/acs.jmedchem.6b00219 26967152

[93] Rubinchik E, Schneider T, Elliott M, Scott WRP, Pan J, Anklin C, Yang H, Dugourd D, Müller A, Gries K, Straus SK, Sahl HG, Hancock REW. 2011. Mechanism of action and limited cross-resistance of new lipopeptide MX-2401. Antimicrob Agents Chemother 55:2743–2754. doi:10.1128/AAC.00170-11 21464247 PMC3101398

[94] Siewert G, Strominger JL. 1967. Bacitracin: an inhibitor of the dephosphorylation of lipid pyrophosphate, an intermediate in the biosynthesis of the peptidoglycan of bacterial cell walls. Proc Natl Acad Sci USA 57:767–773. doi:10.1073/pnas.57.3.767 16591529 PMC335574

[95] Hariguchi N, Chen X, Hayashi Y, Kawano Y, Fujiwara M, Matsuba M, Shimizu H, Ohba Y, Nakamura I, Kitamoto R, Shinohara T, Uematsu Y, Ishikawa S, Itotani M, Haraguchi Y, Takemura I, Matsumoto M. 2020. OPC-167832, a novel carbostyril derivative with potent antituberculosis activity as a DprE1 inhibitor. Antimicrob Agents Chemother 64:e02020-19. doi:10.1128/AAC.02020-19 32229496 PMC7269503

[96] Makarov V, Lechartier B, Zhang M, Neres J, van der Sar AM, Raadsen SA, Hartkoorn RC, Ryabova OB, Vocat A, Decosterd LA, Widmer N, Buclin T, Bitter W, Andries K, Pojer F, Dyson PJ, Cole ST. 2014. Towards a new combination therapy for tuberculosis with next generation benzothiazinones. EMBO Mol Med 6:372–383. doi:10.1002/emmm.201303575 24500695 PMC3958311

[97] Huber J, Donald RGK, Lee SH, Jarantow LW, Salvatore MJ, Meng X, Painter R, Onishi RH, Occi J, Dorso K, Young K, Park YW, Skwish S, Szymonifka MJ, Waddell TS, Miesel L, Phillips JW, Roemer T. 2009. Chemical genetic identification of peptidoglycan inhibitors potentiating carbapenem activity against methicillin-resistant Staphylococcus aureus. Chem Biol 16:837–848. doi:10.1016/j.chembiol.2009.05.012 19716474

[98] Fang X, Tiyanont K, Zhang Y, Wanner J, Boger D, Walker S. 2006. The mechanism of action of ramoplanin and enduracidin. Mol Biosyst 2:69–76. doi:10.1039/b515328j 16880924

[99] de Leeuw E, Li C, Zeng P, Li C, Diepeveen-de Buin M, Lu W-Y, Breukink E, Lu W. 2010. Functional interaction of human neutrophil peptide-1 with the cell wall precursor lipid II. FEBS Lett 584:1543–1548. doi:10.1016/j.febslet.2010.03.004 20214904 PMC3417325

[100] Chamakura KR, Sham LT, Davis RM, Min L, Cho H, Ruiz N, Bernhardt TG, Young R. 2017. A viral protein antibiotic inhibits lipid II flippase activity. Nat Microbiol 2:1480–1484. doi:10.1038/s41564-017-0023-4 28894177 PMC5764540

[101] Lee W, Schaefer K, Qiao Y, Srisuknimit V, Steinmetz H, Müller R, Kahne D, Walker S. 2016. The mechanism of action of lysobactin. J Am Chem Soc 138:100–103. doi:10.1021/jacs.5b11807 26683668 PMC4817722

[102] Hover BM, Kim S-H, Katz M, Charlop-Powers Z, Owen JG, Ternei MA, Maniko J, Estrela AB, Molina H, Park S, Perlin DS, Brady SF. 2018. Culture-independent discovery of the malacidins as calcium-dependent antibiotics with activity against multidrug-resistant Gram-positive pathogens. Nat Microbiol 3:415–422. doi:10.1038/s41564-018-0110-1 29434326 PMC5874163

[103] Ruzin A, Singh G, Severin A, Yang Y, Dushin RG, Sutherland AG, Minnick A, Greenstein M, May MK, Shlaes DM, Bradford PA. 2004. Mechanism of action of the mannopeptimycins, a novel class of glycopeptide antibiotics active against vancomycin-resistant Gram-positive bacteria. Antimicrob Agents Chemother 48:728–738. doi:10.1128/AAC.48.3.728-738.2004 14982757 PMC353120

[104] Brötz H, Bierbaum G, Leopold K, Reynolds PE, Sahl HG. 1998. The lantibiotic mersacidin inhibits peptidoglycan synthesis by targeting lipid II. Antimicrob Agents Chemother 42:154–160. doi:10.1128/AAC.42.1.154 9449277 PMC105472

[105] Smith L, Hasper H, Breukink E, Novak J, Cerkasov J, Hillman JD, Wilson-Stanford S, Orugunty RS. 2008. Elucidation of the antimicrobial mechanism of mutacin 1140. Biochemistry 47:3308–3314. doi:10.1021/bi701262z 18266322

[106] Hsu S-TD, Breukink E, Tischenko E, Lutters MAG, de Kruijff B, Kaptein R, Bonvin AMJJ, van Nuland NAJ. 2004. The nisin-lipid II complex reveals a pyrophosphate cage that provides a blueprint for novel antibiotics. Nat Struct Mol Biol 11:963–967. doi:10.1038/nsmb830 15361862

[107] Schneider T, Kruse T, Wimmer R, Wiedemann I, Sass V, Pag U, Jansen A, Nielsen AK, Mygind PH, Raventós DS, Neve S, Ravn B, Bonvin AMJJ, De Maria L, Andersen AS, Gammelgaard LK, Sahl H-G, Kristensen H-H. 2010. Plectasin, a fungal defensin, targets the bacterial cell wall precursor Lipid II. Science 328:1168–1172. doi:10.1126/science.1185723 20508130

[108] Shukla R, Lavore F, Maity S, Derks MGN, Jones CR, Vermeulen BJA, Melcrová A, Morris MA, Becker LM, Wang X, et al.. 2022. Teixobactin kills bacteria by a two-pronged attack on the cell envelope. Nature 608:390–396. doi:10.1038/s41586-022-05019-y 35922513 PMC9365693

[109] Lee K, Campbell J, Swoboda JG, Cuny GD, Walker S. 2010. Development of improved inhibitors of wall teichoic acid biosynthesis with potent activity against Staphylococcus aureus. Bioorg Med Chem Lett 20:1767–1770. doi:10.1016/j.bmcl.2010.01.036 20138521 PMC2844852

[110] Matano LM, Morris HG, Hesser AR, Martin SES, Lee W, Owens TW, Laney E, Nakaminami H, Hooper D, Meredith TC, Walker S. 2017. Antibiotic that inhibits the ATPase activity of an ATP-binding cassette transporter by binding to a remote extracellular site. J Am Chem Soc 139:10597–10600. doi:10.1021/jacs.7b04726 28727445 PMC5699463

[111] Thompkins K, Chattopadhyay B, Xiao Y, Henk MC, Doerrler WT. 2008. Temperature sensitivity and cell division defects in an Escherichia coli strain with mutations in yghB and yqjA, encoding related and conserved inner membrane proteins. J Bacteriol 190:4489–4500. doi:10.1128/JB.00414-08 18456815 PMC2446817

[112] Boughner LA, Doerrler WT. 2012. Multiple deletions reveal the essentiality of the DedA membrane protein family in Escherichia coli. Microbiology (Reading) 158:1162–1171. doi:10.1099/mic.0.056325-0 22301910

[113] Doerrler WT, Sikdar R, Kumar S, Boughner LA. 2013. New functions for the ancient DedA membrane protein family. J Bacteriol 195:3–11. doi:10.1128/JB.01006-12 23086209 PMC3536176

[114] Roney IJ, Rudner DZ. 2024. Bacillus subtilis uses the SigM signaling pathway to prioritize the use of its lipid carrier for cell wall synthesis. PLoS Biol 22:e3002589. doi:10.1371/journal.pbio.3002589 38683856 PMC11081497

[115] Eiamphungporn W, Helmann JD. 2008. The Bacillus subtilis σ^M^ regulon and its contribution to cell envelope stress responses. Mol Microbiol 67:830–848. doi:10.1111/j.1365-2958.2007.06090.x 18179421 PMC3025603

[116] Roney IJ, Rudner DZ. 2025. Two new enzymes that liberate undecaprenyl-phosphate to replenish the carrier lipid pool during envelope stress. mBio 16:e0371024. doi:10.1128/mbio.03710-24 39878533 PMC11898649

[117] Marmont LS, Orta AK, Baileeves BWA, Sychantha D, Fernández-Galliano A, Li YE, Greene NG, Corey RA, Stansfeld PJ, Clemons WM Jr, Bernhardt TG. 2024. Synthesis of lipid-linked precursors of the bacterial cell wall is governed by a feedback control mechanism in Pseudomonas aeruginosa. Nat Microbiol 9:763–775. doi:10.1038/s41564-024-01603-2 38336881 PMC10914600

[118] Penterman J, Nguyen D, Anderson E, Staudinger BJ, Greenberg EP, Lam JS, Singh PK. 2014. Rapid evolution of culture-impaired bacteria during adaptation to biofilm growth. Cell Rep 6:293–300. doi:10.1016/j.celrep.2013.12.019 24412364 PMC3941072

[119] Ftácek P, Skultéty L, Toman R. 2000. Phase variation of Coxiella burnetii strain Priscilla: influence of this phenomenon on biochemical features of its lipopolysaccharide. J Endotoxin Res 6:369–376. doi:10.1177/09680519000060050701 11521057

[120] Beare PA, Jeffrey BM, Long CM, Martens CM, Heinzen RA. 2018. Genetic mechanisms of Coxiella burnetii lipopolysaccharide phase variation. PLoS Pathog 14:e1006922. doi:10.1371/journal.ppat.1006922 29481553 PMC5843353

[121] Long CM, Beare PA, Cockrell D, Binette P, Tesfamariam M, Richards C, Anderson M, McCormick-Ell J, Brose M, Anderson R, Omsland A, Pearson T, Heinzen RA. 2024. Natural reversion promotes LPS elongation in an attenuated Coxiella burnetii strain. Nat Commun 15:697. doi:10.1038/s41467-023-43972-y 38267444 PMC10808227

